# Contextualized Design of IoT (Internet of Things) Finance for Edge Artificial Intelligence Computing

**DOI:** 10.1155/2022/6046957

**Published:** 2022-03-09

**Authors:** Yixuan Guo

**Affiliations:** Business Administration, Shanghai Lixin University of Accounting and Finance, Shanghai 201209, China

## Abstract

With the widespread application of IoT technology in the world, the new industry of IoT finance has emerged. Under this new business model, commercial banks and other financial institutions can realize safer and more convenient financial services such as payment, financing and asset management through the application of IoT technology and communication network technology. In the cloud computing model, the local terminal device of IOT will transmit the collected data to the cloud server through the network, and the cloud server will complete the data operation. Cloud computing model can well solve the problem of poor performance of IoT devices, but with the increasing number of IoT terminal devices and huge number of devices accessing the network, cloud computing model is constrained by network bandwidth and performance bottleneck, which brings a series of problems such as high latency, poor real-time and low security. In this paper, based on the new industry of IoT finance which is developing rapidly, we construct a POT (Peaks Over Threshold) over threshold model to empirically analyze the operational risk of commercial banks by using the risk loss data of commercial banks, and estimate the corresponding ES values by using the control variables method to measure the operational risk of traditional commercial banks and IoT finance respectively, and compare the total ES values of the two. This paper adopts the control variable method to reduce the frequency of each type of loss events of operational risk of commercial banks in China respectively.

## 1. Introduction

The level of management of operational risk in commercial banks has improved compared to the previous level, but it is still not perfect, so that cases of losses due to operational risk occur from time to time and cause losses to commercial banks [1]. The transformation of IoT technology for finance is limited, which leads to the fact that no scholars abroad have conducted segmented research on IoT finance [2]. As a new industry emerged in recent years, there is a lack of research on risk management in IoT finance industry in China, and the research conducted by scholars in this area is mainly based on the risk of supply chain financing, risk of movable pledge financing and the reconstruction of risk management system of commercial banks in IoT finance industry [3]. With the emergence of the new industry of IoT finance, the risk management of commercial banks will be reconstructed, and commercial banks will realize the risk control thinking through IoT finance [4]. The IoT finance will enable commercial banks to achieve objective risk control thinking, technical risk control tools, data-based risk control processes and accurate risk control decisions. Therefore, commercial banks should actively layout IoT finance to realize the “four” reconfiguration of bank risk as soon as possible [5].

Image processing can be combined with a variety of application scenarios, and has huge application space in various fields. In the field of intelligent medical treatment, image processing is applied to medical imaging intelligent analysis, auxiliary medical diagnosis, etc [6]. It can be used for tumor detection and tumor development analysis; in the field of intelligent security, it is applied to license plate recognition and skynet monitoring system deployment, etc.; in the field of intelligent driving, it is applied to image acquisition, object detection and recognition, etc. In daily life, the application of image processing is also closely related to us, among which face detection and recognition is the most widely used in daily life. Through face detection technology, interesting applications such as AI face changing and tracking beauty can be realized, and through face recognition technology, face payment, face login App, face check-in and face crossing gates can be directly swiped [7]. In addition to face detection and recognition, image recognition and analysis is also a more widely used scenario for image processing, through which image recognition and analysis can be used to identify car models, fruit types and pests, etc [8]. Some common functions of cell phones, such as Taobao photo shopping function “Patlite” and automatic classification of cell phone albums, are also inseparable from image processing [9]. The support of image processing technology is also indispensable. Image processing combined with artificial intelligence technology can greatly improve the speed of computing, reduce the cost of processing operations, and improve the accuracy of image recognition. Edge computing refers to the use of a platform that integrates data computing, data storage, network transmission and other application core capabilities on the side close to data collection to provide local near-end services for data processing [10]. After the data processing is completed, the edge side can send the useful data to the cloud server. The data processing method corresponding to edge computing is cloud computing [11]. Cloud computing means that after completing data collection, the data is transmitted to the cloud server through network transmission, etc., and then the cloud completes data processing and returns the results [12].

The purpose of this paper is to study the operational risk of commercial banks in the IoT financial industry. IoT finance is a new financial industry that emerged with the rapid development of IoT technology and its large-scale application. IoT finance has changed the traditional financial industry to collect information in the way of competent verification and promote the financial industry to achieve objective verification of the whole process. payment, financing, asset management and other financial services. IoT finance will bring new ideas for commercial banks' operational risk management. In this paper, we collect the loss data of commercial banks from 2010 to 2017 from public sources for each type of operational risk, and by constructing the extreme value theory model POT over threshold model and combining with the control variables method, we measure and empirically analyze the operational risk of traditional commercial banks and commercial banks in the IoT financial industry, and calculate the corresponding VaR and ES values respectively to compare the operational risk of traditional commercial banks and IoT financial industry. The results show that the latter can effectively reduce the operational risk of commercial banks, so commercial banks should vigorously implement the deployment and application of IoT technology and actively carry out IoT financial business.

## 2. Related Work

Due to the strict regulation of foreign financial environment, the transformation of IoT technology for finance is restricted, which leads to no scholars in foreign countries to conduct segmented research on IoT finance [13]. As a new industry emerged in recent years, there is a lack of research on risk management in IoT finance industry in China, and the research conducted by scholars in this area is mainly based on the risk of supply chain financing, risk of movable pledge financing and the reconstruction of risk management system of commercial banks in IoT finance industry. Regarding the risk of supply chain financing under the IoT financial mode, the credit risk and operational risk under the traditional supply chain financing operation stage and process are extracted, and the new model of supply chain financing under the IoT financial mode is proposed by Mauthe et al., which will effectively reduce the above supply chain financing risk, but at the same time, IoT finance as a new industry will certainly bring new risks, according to the IoT [14]. According to the IoT system architecture, the new risks faced by supply chain financing under IoT finance mode are divided into perception layer risk, network layer risk and application layer risk, so the control of new risks needs to be considered from the implementation of IoT finance related technical standards and specifications. Regarding the risk of chattel pledge financing, they created the IOT chattel financing system, which is to crack the traditional chattel financing problem by further reducing the risk of chattel pledge financing effectively, using the advantage of objective verification of IOT finance itself to “immobilize” the chattel and promote the change of industry credit system to objective credit system [15]. The movable property financing system created based on IOT financial mode can be divided into three levels, namely, IOT finance, IOT intelligent network and IOT warehouse receipt.

Crowd information detection is an important part of urban security and modern city management field. With the development of the city, urban population density is also rising, public places in all kinds of entertainment activities and public activities are increasing. Chen et al. proposed that the safety of dense crowds of people has become a very serious problem [16]. At home and abroad, there have been many cases of accidents due to overly dense crowds, trampling on each other and causing casualties. Establishing a real-time crowd information detection system can analyze the abnormal conditions in the crowd in time, issue early warning information, and notify relevant departments to take necessary actions to manage the crowd order and avoid accidents. In conclusion, the use of effective image analysis technology to achieve quantified pedestrian information data and predict the crowd density of an urban area can provide effective data support for urban security monitoring, public space design, urban planning and resource scheduling, etc [17]. In response to the poor real-time computing performance in the cloud, as well as the high requirements for power consumption, storage and network bandwidth, Ancuti et al. designed an edge computing system based on artificial intelligence image processing, in which crowd information data will be processed at the edge to accelerate the computing of crowd information analysis and save network bandwidth and power consumption [18].

For cloud computing requires real-time image uploading from the edge to the central server for unified analysis and processing, which has poor real-time performance, and also requires high power consumption, storage and network bandwidth for a series of problems, this project proposes a convolutional neural network-based edge computing model for real-time crowd information detection [19]. They proposed that the edge system is the main part of the crowd information detection system design in this thesis [20]. In the edge system, there are multiple data acquisition nodes, and each node camera performs image acquisition, and then the processor of each node performs data arithmetic processing, crowd feature extraction and analysis based on neural network model to get the number of crowd at that node. Each node sends data logs through the network to the regional summary point, which performs data cleaning and aggregation, and performs simple analysis and alerting, such as the total number of people in the region, analysis of crowd flow direction in the region and alerting of crowding level at each node [21]. The regional summary point aggregates the data of this region into logs and sends them to the cloud server through the network. After receiving the message, the cloud server can perform further in-depth analysis, visualization processing and intelligent decision on the data, and make backups of the historical data.

## 3. Edge Computing for Financial Scenario Design

### 3.1. Edge Computing System Based on Crowd Information Detection Task

**Figure 1:**
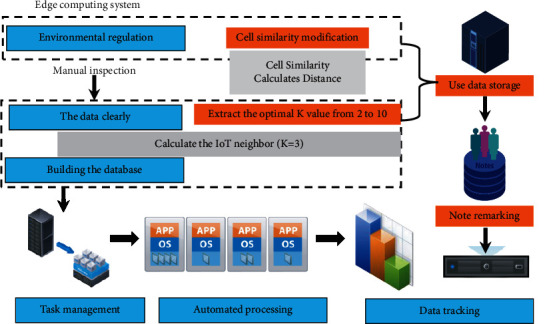
Edge computing system.

In the whole edge computing system, this paper focuses on the edge system implementation part, which implements the deployment of neural network algorithms, image acquisition and processing at the edge, simple analysis and prediction of edge nodes and communication with the cloud server. The specific implementation of the edge computing system is shown in Figure 1.

The training of the convolutional neural network model is completed on the workstation. This project refers to the structural design of the CRSnet model and makes targeted improvements based on the poor computing power of the processor at the edge. The main optimizations include.

1. The CSRnet network model is replaced from the vggnet model to the lightweight mobilenet model. mobilenet is a lightweight convolutional neural network suitable for deployment at the edge where performance is relatively low, and the computational effort is greatly reduced by model replacement.
2. Parameter pruning was performed on the model. By evaluating the algorithm, the unimportant parameters and redundant channels are trimmed during the training process to reduce the amount of network operations while ensuring accuracy.
3. Quantization of parameters is performed. The parameter quantization pair converts the original 32 bit floating-point weights and activation values to 8 bit fixed-point values. The quantized 8 bit fixed-point parameters not only reduce the storage space of the parameters, but also require less resources in hardware and have higher running speed compared to floating-point operations. The model compression is performed by model channel trimming and parameter quantization, and the number of parameters is reduced to 6.67% of the original model. The compressed model is exported to the embedded platform in tensorflow lite format.

In the edge computing model, the artificial intelligence neural network algorithm is first deployed into the edge device, and the edge device collects the image or video information of the crowd in the area through the camera module in real time, while processing and analyzing the collected image or video information using the neural network algorithm, and uploading the structured crowd information to the cloud center. The data from multiple Raspberry Pi nodes are aggregated to the ARC development board to complete the aggregation and analysis of crowd information and prediction, etc. The system functions include data cleaning, data aggregation, crowd crowd alert, crowd direction estimation, etc. The crowd information detection edge system can process and aggregate crowd information data 12 times per minute, and the error is less than 18% on the test data set, which can meet the requirements of crowd information detection. This chapter accomplishes the crowd information detection task using an edge computing platform for artificial intelligence image processing.

### 3.2. Financial Scenario-Based Design and Operational Risk Analysis

In this paper, according to the classification of operational risk by Basel Committee on Banking Supervision (BCBS), combined with the application of IoT technology in commercial banks under the current IoT financial industry, the operational risk of commercial banks is divided into: internal fraud, external fraud, loss and damage of pledges, risk of operational errors of practitioners and risk of IoT system generated by the new IoT financial industry. Internal fraud risk mainly refers to bank employees who cannot resist the temptation of profit, accepting bribes or kickbacks, colluding with external unscrupulous people, colluding with each other, turning a blind eye to the business that fails to meet the borrowing requirements or deliberately conceals the hidden risks, which makes the bank suffer huge capital losses and seriously damages the integrity image of the bank and the banking industry as a whole. The system model of edge computing is designed in this chapter, using Raspberry Pi as the edge end, and the model is optimized by pruning and parameter quantization of the convolutional neural network to reduce the computation and adapt to the edge computing model, and the convolutional neural network is deployed to the Raspberry Pi development board to complete crowd image acquisition and crowd information extraction.

Suppose *X* represents the gain or loss of a certain portfolio, and *a* represents the gain of the aforementioned portfolio when *X* > 0 at a certain confidence level, and the loss of the investment when *X* < 0. VaR is the upper limit of the possible loss of the portfolio in the investment, and the equation about VaR is shown in the formula:

(1) $$\begin{array}{c} P_{\text{VaR}}\left(x\right)=\alpha ,\;x<V. \end{array}$$

The VaR model is now widely used in the financial sector, not only for the overall measurement of different risks using a uniform measurement method, but also for the control of risks in each sector or business through risk limits. However, in the process of applying VaR model in commercial banks, the shortcomings of the model have been found. Since it is essentially a quantile at a certain confidence level, it is still inadequate for tail risk consideration because it is impossible to measure the left tail risk beyond the quantile and it is easy to ignore small probability extreme events. In addition, the overall risk is not necessarily smaller than the sum of individual risks, which is contrary to risk diversification. The relationship between ES and VaR is shown in Eq:

(2) $$\begin{array}{c} \text{ES}\left(x\right)=P\left[-x,x>\text{VaR}\right]. \end{array}$$

An optimal threshold is selected, and then a new dataset is constructed for the data that exceeds this threshold, and then this dataset is modeled. Assume that *X*_1_, *X*_2_,…, *X*_*n*_ denote the sample data of each type of loss events of commercial banks' operational risk, and use threshold selection tools, such as the excess mean function graph method and kurtosis method, to select reasonable thresholds for each type of operational risk loss data respectively. Assuming that *X* is selected as the optimal threshold *u*, the conditional distribution function exceeding the optimal threshold can be derived, as shown in equation:

(3) $$\begin{array}{c} F\left(x\right)=P\left(\frac{X-\alpha }{u}>y,x>\text{VaR}\right). \end{array}$$

The derivation of the above distribution function using the conditional probability formula yields:

(4) $$\begin{array}{c} F\left(y\right)=\frac{F\left(x\right)+F\left(x+1\right)}{1-F\left(x\right)},\\ x=\frac{y-\alpha }{u}. \end{array}$$

The expression for the overall distribution function is obtained by the derivative transformation:

(5) $$\begin{array}{c} F_{n}\left(x\right)=F\left(y\right)\left(1-F\left(x\right)\right),\\ x=\frac{y-\alpha }{u}. \end{array}$$

**Figure 2:**
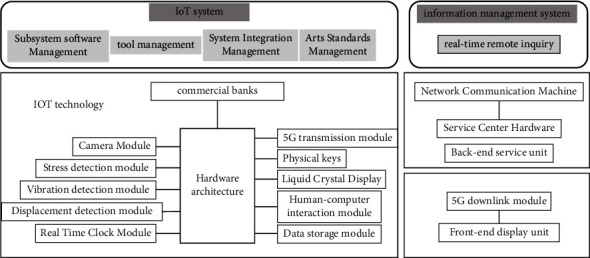
Internal scenario design for financial management.

Internal fraud cases occur repeatedly, on the one hand, because it is about the professional conduct of practitioners, internal management system and process optimization is still based on the setting and reliance on personnel, moral hazard can not be completely avoided; on the other hand, the bank to “people management” thinking as the leading logic of internal management thinking, it is difficult to fundamentally eliminate internal fraud On the other hand, the internal management logic of banks, which is dominated by the “human management” mindset, can hardly prevent internal fraud from occurring. The commercial banks in the IOT financial industry use the IOT technology to transform and upgrade the banks, replacing the previous “human management” thinking logic of commercial banks with the “management of things” and “management of people” thinking logic, thus effectively reduce the risk of fraud within commercial banks, as shown in Figure 2.

With the development of IOT technology, banks can connect financing enterprises and third-party logistics supervision enterprises through IOT technology, fully grasp the information of the whole process from procurement to sales of financing enterprises, real-time objective investigation and loan approval of financing enterprises, effectively avoiding the occurrence of external fraud risk events. In addition, IOT technology will also promote the change of movable financing, for the joint fraud of third-party logistics enterprises and financing enterprises resulting in false billing and duplicate pledges, can promote the formation of unique and exclusive characteristics of the Internet of Things warehouse receipt, replacing the traditional receipt or inventory list, to solve the problem of duplicate pledges, false warehouse receipts, etc., to further reduce the risk of external fraud. Through real-time monitoring of crowd information, abnormal conditions appearing in regional crowds can be detected in time and some illegal activities can be found in time.

**Figure 3:**
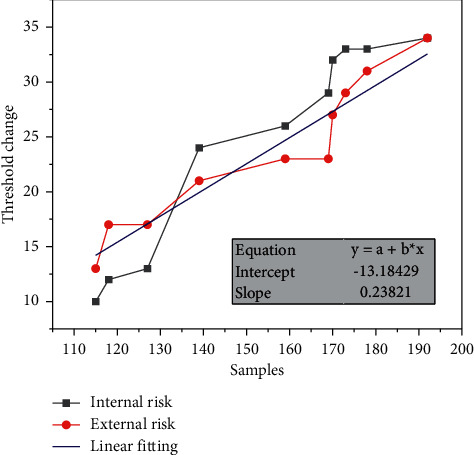
Excess threshold change.

It can be said that the key to the success of the POT model lies in whether the selection of the threshold is reasonable or not. For the selection of the threshold, too large or too small will affect the accuracy of the final measurement results, and thus the prediction of operational risk accrual of commercial banks will be biased. Therefore, when selecting the optimal threshold, scholars often use multiple threshold selection methods to select multiple thresholds or a certain method to select multiple thresholds, and then determine an optimal threshold from these thresholds. In this paper, we adopt the latter paradigm of determining the optimal threshold by using the excess mean function graph method to select multiple thresholds and then determine the final optimal threshold from them, as shown in Figure 3.

1. By using Matlab software to draw the excess mean function graph for each type of loss event of commercial banks' operational risk, the excess function mean graph is observed, and when there is an obvious linear change from a certain point, the value of *u* corresponding to that point is the initially selected threshold, so the selected threshold is affected by individual subjectivity.
2. In view of this, in this paper, three thresholds *u*_1_, *u*_2_, and *u*_3_ will be initially selected using the excess function mean value plot, and then the above initially selected thresholds will be substituted into the log-likelihood function, and the parameters *θ* and *δ* corresponding to each threshold will be estimated by great likelihood estimation.
3. Then the corresponding *χ*^2^ values will be calculated and a chi-square goodness-of-fit test will be performed to determine the optimal thresholds for each type of loss event of operational risk.

### 3.3. Value-at-Risk of IoT Financial Operations

**Figure 4:**
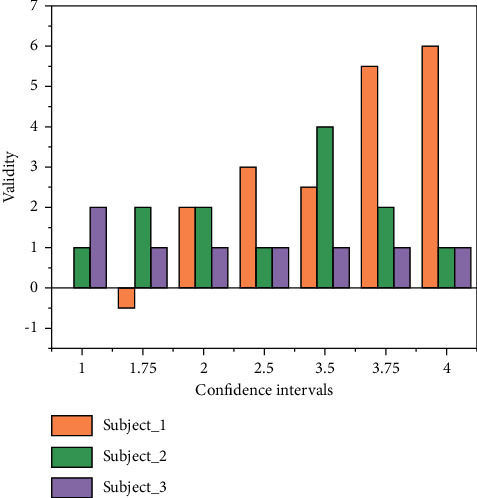
Correlation of validity with confidence intervals.

VaR (Value at Risk), which is simply the value at risk, i.e., the value at risk, is the maximum loss that could be incurred by a financial asset or portfolio under normal market conditions. The use of VaR model should follow the assumption of market effectiveness, and market fluctuations should be random, but in reality, the effectiveness of the market in most countries are relatively weak, which requires scholars to do an approximate normal treatment to maximize the use of VaR analysis tools when the market has incomplete satisfaction of the effectiveness and fluctuations of randomness. In the definition of VaR, holding period and confidence level are two very important parameters of VaR. Only when the parameters are given, the construction of VaR model will be valuable. Considering that the portfolio return does not necessarily obey normal distribution, generally for the holding period is chosen from short, the shorter the holding period, the more the return approximately obeys normal distribution. The choice of confidence level is very critical. The attitude of the investment subject towards risk can be reflected from the confidence level, and a higher confidence level corresponds to a greater degree of risk aversion. In addition, the choice of confidence level depends on the specific purpose, such as to consider the effectiveness of VaR at subject_1, subject_2 and subject_3, it is necessary to choose a lower confidence level, if the results are to be compared, a higher confidence level is often more appropriate, as shown in Figure 4.

Based on the determined reasonable threshold *u*, the data in the operational risk loss data set that exceeds this threshold is constructed into a new data set, assuming that the new data set is *y*(*y*_1_, *y*_2_,…, *y*_*t*_), in order for *y*(*y*_1_, *y*_2_,…, *y*_*t*_) obey the GPD distribution as much as possible, this requires that when choosing a reasonable threshold, a sufficiently large threshold is selected as much as possible, and the formula expresses that at a sufficiently large threshold *u*, the new data set *y*(*y*_1_, *y*_2_,…, *y*_t_) obeys the GPD distribution:

(6) $$\begin{array}{c} F\left(x\right)=\left\{\begin{array}{c} F_{1}=\frac{f_{11}}{\left\|f_{11}\right\|},\frac{f_{12}}{\left\|f_{12}\right\|},\\ F_{2}=\frac{f_{1}}{\left\|f_{1}\right\|},\frac{f_{2}}{\left\|f_{2}\right\|},\\ F=\left[F1,F2\right]. \end{array}\right. \end{array}$$

Substituting *y* = *x* − *u* into equations yields the expression for the generalized Pareto distribution, where *δ* is the shape parameter and *θ* is the scale parameter:

(7) $$\begin{array}{c} F\left(x\right)=\left\{\begin{array}{c} \tan\left(\frac{e^{x}-e^{-x}}{e^{x}+e^{-x}}\right),\;x=1,\\ 1-e^{x},\;x>1. \end{array}\right. \end{array}$$

When the crowd density exceeds the set alert line, an early warning is made to notify the relevant departments to take necessary actions to maintain the order of the scene and evacuate the crowd in order to prevent a collective incident. To derive the expression for the log-likelihood function, the probability density function can be derived by first deriving the expression for the generalized Pareto distribution as follows:

(8) $$\begin{array}{c} g\left(x\right)=\max\left(e^{x}-e^{-x}\right). \end{array}$$

The results are shown in equation:

(9) $$\begin{array}{c} \text{VaR}\left(\alpha \right)=\left\{\begin{array}{c} C_{t}\left\{t_{1},t_{2},\ldots t_{i}\right\},\\ \left(1+\alpha \right)\frac{q\left[c_{s},w\right]}{t}. \end{array}\right. \end{array}$$

## 4. Experiments and Results Analysis

### 4.1. Descriptive Statistical Analysis

**Figure 5:**
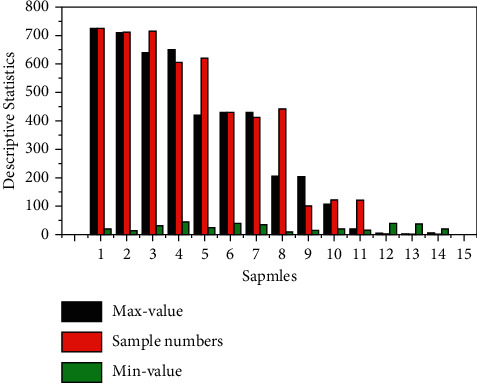
Descriptive statistics of operational risk loss events.

In this paper, in order to facilitate the subsequent comparative analysis, the operational risk loss events are mainly divided into four categories, and the EViews software is used to conduct descriptive statistical analysis of each type of loss data, and the analysis results are shown in Figure 5. According to the results, it can be found that the skewness of each type of loss events of operational risk in Chinese commercial banks is greater than 0, and the kurtosis is greater than 3, which indicates that the loss sample data of all types of loss events of commercial banks show a thick-tailed distribution, and also show the characteristics of low frequency and high loss of operational risk events. The results show that commercial banks in the IoT financial industry can effectively reduce operational risk, which leads to the conclusion that commercial banks should vigorously implement the deployment and application of IoT technology, and improve the level of control of operational risk with the help of the new industry of IoT finance, combining with the actual situation of banks themselves to minimize the operational risk and reduce the losses of banks.

### 4.2. Scenario-Based Design and Operational Risk Measurement

**Figure 6:**
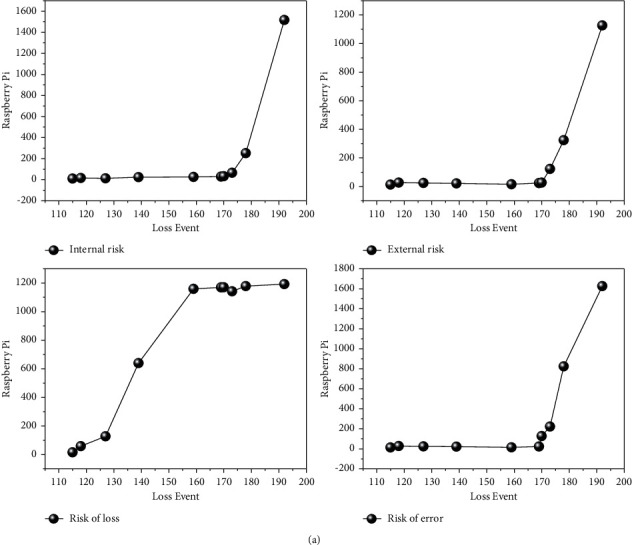
Operational risk change curve by type of loss event.

In this paper, we design an edge computing system for crowd information detection, and train an optimized convolutional neural network to deploy the network on a Raspberry Pi development board to accomplish the task of crowd information detection based on convolutional neural network and edge computing. Crowd information detection, including crowd counting, crowd direction analysis and crowd behavior analysis, is an important part of urban security and modern city management. In this chapter, the system model of edge computing is designed, and the Raspberry Pi is used as the edge end. Through the optimization of the convolutional neural network such as pruning and parameter quantization, the model reduces the computation and adapts to the edge computing model, and the convolutional neural network is deployed to the Raspberry Pi development board to complete crowd image acquisition and crowd information extraction. In order to further verify the accuracy of the thick-tailed distribution of the sample data, the plots of each type of sample data were drawn by Matlab software, as shown in Figure 6, and it can be found that the data points in the plots are all curved upward, which indicates that the data obey the thick-tailed distribution.

**Figure 7:**
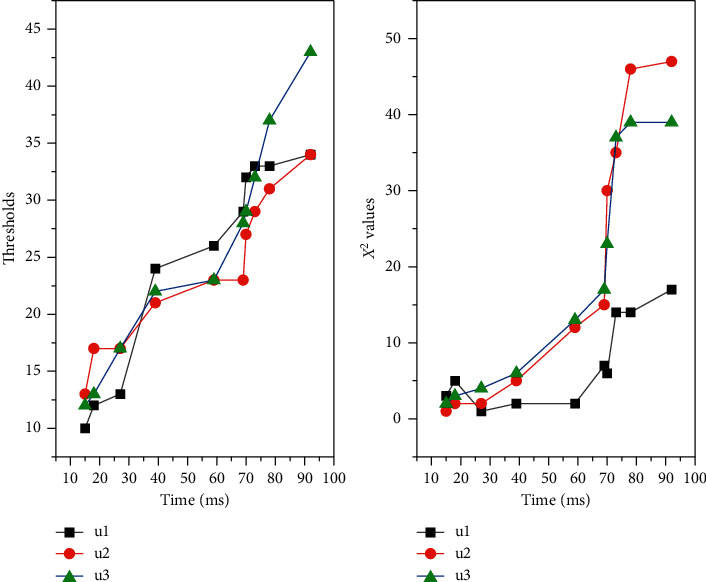
Thresholding and parameter estimates.

This paper adopts the control variable method to reduce the frequency of each type of loss events of operational risk of commercial banks in China respectively. By observing the mean value plot of the excess function, when there is an obvious linear change from a certain point, the *u* value corresponding to that point is the preliminary selected threshold. By this method, the author initially selected the thresholds *u*_1_, *u*_2_, and *u*_3_ for each type of loss event, in which the preliminary selected thresholds for internal fraud risk are: *u*_1_ = 4990, *u*_2_ = 16209, and *u*_3_ = 54000; the preliminary selected thresholds for external fraud risk are: *u*_1_ = 1500, *u*_2_ = 2922, and *u*_3_ = 5765; the preliminary selected thresholds for pledge loss damage risk are: *u*_1_ = 1142, *u*_2_ = 2000, and *u*_3_ = 5765. The initial thresholds for the risk of external fraud are: *u*_1_ = 1500, *u*_2_ = 2922, *u*_3_ = 5765; the initial thresholds for the risk of loss and damage of pledges are: *u*_1_ = 1142, *u*_2_ = 2000, *u*_3_ = 2640; the initial thresholds for the risk of operator error are: *u*_1_ = 170, *u*_2_ = 332, *u*_3_ = 1500. then the above initially selected thresholds are substituted into it. Then, the above selected thresholds are substituted into the log-likelihood function, and the parameters *θ* and *δ* corresponding to each threshold are estimated with great likelihood, and then the corresponding *χ*^2^ values are calculated and the chi-square goodness-of-fit test is performed to select the optimal thresholds, as shown in Figure 7.

**Figure 8:**
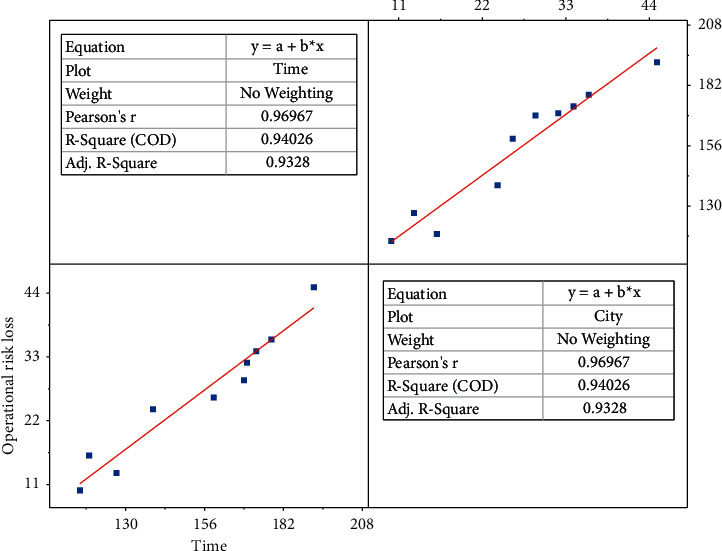
Descriptive statistical analysis of operational risk loss events in commercial banks in the IoT financial sector.

IoT thinking and technology can make up for the limitations and pain points of traditional risk management system of commercial banks in China to the greatest extent, and commercial banks can effectively reduce the number of occurrence of each type of loss events of operational risk through IoT transformation of bank branches and IoT upgrading of bank business processes, etc. The frequency of the occurrence of each type of loss events of operational risk of commercial banks in China is reduced by using the control variables method, while the data of losses caused by the IoT system risk brought by the new industry of IoT finance is increased, and the data of losses caused by the IoT system risk of commercial banks are collected through the website of adjudication documents and some portals, and the data of losses caused by the IoT system risk are adjusted accordingly. Then descriptive statistical analysis was conducted on the data of commercial banks' operational risk losses under the IoT financial industry, and the results are shown in Figure 8.

By observing the mean value plot of the excess function, when there is an obvious linear change from a certain point, the *u* value corresponding to that point is the preliminary selected threshold. By this method, the author initially selected the thresholds *u*_1_, *u*_2_, and *u*_3_ for each type of loss event, in which the preliminary selected thresholds for internal fraud risk are: *u*_1_ = 4990, *u*_2_ = 10000, and *u*_3_ = 56000; the preliminary selected thresholds for external fraud risk are: *u*_1_ = 1000, *u*_2_ = 2922, and *u*_3_ = 5765; the preliminary selected thresholds for pledge loss damage risk are: *u*_1_ = 750, *u*_2_ = 1142, and *u*_3_ = 5765. The initial thresholds for the risk of external fraud are: *u*_1_ = 1000, *u*_2_ = 2922, *u*_3_ = 5765; the initial thresholds for the risk of loss and damage of pledged items are: *u*_1_ = 750, *u*_2_ = 1142, *u*_3_ = 2640; the initial thresholds for the risk of operator error are: *u*_1_ = 90, *u*_2_ = 160, *u*_3_ = 332; the initial thresholds for the risk of IoT system are: *u*_1_ = Then, the above initial selected thresholds are substituted into the log-likelihood function, and the great likelihood estimation is performed to obtain the parameters *θ* and *δ* corresponding to each threshold, and then the corresponding *χ*^2^ values are calculated and the chi-square goodness-of-fit test is performed to select the optimal threshold.

## 5. Conclusion

In this paper, based on the new industry of IoT finance, we construct a POT over threshold model to analyze the operational risk of commercial banks by using the data of operational risk loss of banks, and estimate the corresponding ES value by using the control variables method to measure the operational risk of traditional commercial banks and IoT finance respectively, and compare the total ES value of both. In this paper, we design a RISC-V convolution acceleration instruction based on the 4 × 4 feature map of Winograd algorithm and the convolution operation of 3 × 3 convolution kernel as a convolution acceleration operator. With this acceleration instruction, the convolution operation of 4 × 4 feature map with 3 × 3 convolution kernels is implemented with about 77.8% less memory access than before the optimization, and the performance is improved by more than 5 times, and the acceleration performance is improved to 11.7 times after the memory access optimization, and the performance will be improved even more under the condition that the activation function is ReLU and specific pooling operation. In addition, the coprocessor acceleration module can use the 4 × 4 feature map based on Winograd algorithm with 3 × 3 convolution kernel as a Winograd acceleration operator to perform convolution operation on feature map chunks with Winograd acceleration operator, which also has excellent performance.

## Data Availability

The data used to support the findings of this study are available from the corresponding author upon request.
